# Effect of hydrocortisone on interleukin-6 production in human ,peripheral blood rnononuclear ceils

**DOI:** 10.1155/S0962935192000036

**Published:** 1992

**Authors:** Y. Hirooka, T. Mitsuma, T. Nogimori, Y. Ishizukis

**Affiliations:** 1 The Fourth Department of Internal Medicine Aichi Medical University Yazako Nagakute-cho, Aichi-gun, Aichi-ken 480-11 Japan; 2 Department of Medicine Konanshowa Hospital Konan Aichi Japan; 3 Ishizuki Thyroid Clinic Nagoya Japan

## Abstract

The effect of hydrocortisone on the production of interleukin-6 (IL-6) in human peripheral blood mononuclear cells was studied. Using our newly developed radioimmunoassay system for IL-6 of which specificity, reproducibility, sensitivity and usefulness have been demonstrated. IL-6 production in peripheral blood mononuclear cells of ten normal subjects revealed that in lipopolysaccharide (LPS, 10*μ*g/ml)-stimulation, the mean ± SD of IL-6 was 2.71 ± 0.85 ng/ml. No detectable amount of IL-6 was observed in the absence of LPS and in the presence of hydrocortisone alone. Hydrocortisone (10^−10^ M to 10^−3^ M) inhibited LPS-stimulated IL-6 production in a dose-dependent manner. However, there was a wide variation in the response to hydrocortisone, namely, ranging from steroid-sensitive to steroid-resistant. Based on the concentration required to inhibit 50% of LPS-stimulated IL-6 production, three of ten subjects were at 10^−6^ M, three at 10^−5^ M and the rest at 10^−4^ M, respectively. The dramatic anti-inflammatory and immunosuppressive effects of glucocorticosteroids can be life-saving in autoimmune diseases. The present findings suggested that there existed the differences in susceptibility to glucocorticosteroids even among normal subjects, providing some implications for the drug treatment, and also gave further evidence that there may exist an immunoregulatory feedback circuit between the immune and neuroendocrine systems.


Mediators of Inflammation 1, 9–13 (1992)

### Effect of hydrocortisone on interleukin-6 production in human peripheral blood mononuclear cells

Y. Hirooka,[1,CA] T. Mitsuma,[1] T. Nogimori[2] and Y. Ishizuki[3]

[1]The Fourth Department of Internal Medicine, Aichi Medical University, Yazako, Nagakute-cho, Aichi-gun, Aichi-ken, 480-11. Japan;
[2]Department of Medicine, Konanshowa Hospital, Konan, Aichi, Japan;
[3]Ishizuki Thyroid Clinic, Nagoya, Japan.

[CA] Corresponding Author

**THE effect of hydrocortisone on the production of interleukin-6 (IL-6) in human peripheral blood mononuclear cells was studied. Using our newly developed radioimmunoassay system for IL-6 of which specificity, reproducibility, sensitivity and usefulness have been demonstrated. IL-6 production in peripheral blood mononuclear cells of ten normal subjects revealed that in lipopolysaccharide (LPS, 10$\mu$g/ml)-stimulation, the mean $\pm$SD of IL-6 was 2.71 $\pm$0.85 ng/ml. No detectable amount of IL-6 was observed in the absence of LPS and in the presence of hydrocortisone alone. Hydrocortisone ($10^{-10}$ M to $10^{-3}$ M) inhibited LPS-stimulated IL-6 production in a dose-dependent manner. However, there was a wide variation in the response to hydrocortisone, namely, ranging from steroid-sensitive to steroid-resistant. Based on the concentration required to inhibit 50% of LPS-stimulated IL-6 production, three of ten subjects were at $10^{-6}$ M, three at $10^{-5}$ M and the rest at $10^{-4}$ M, respectively. The dramatic anti-inflammatory and immunosuppressive effects of glucocorticosteroids can be life-saving in autoimmune diseases. The present findings suggested that there existed the differences in susceptibility to glucocorticosteroids even among normal subjects, providing some implications for the drug treatment, and also gave further evidence that there may exist an immunoregulatory feedback circuit between the immune and neuroendocrine systems.**

**Key words:** Culture supernatant, Glucocorticoid, Human peripheral blood mononuclear cell, IL-6 radioimmunoassay, Interleukin-6

### Introduction

Glucocorticoids have assumed a major role in the treatment of numerous autoimmune, allergic and malignant diseases and also for the allograft rejection. It is becoming increasingly clear that lymphocytes and macrophages, as primary effectors of cellular and humoral immune responses, are the focal points for many of the clinically useful properties of glucocorticoids.

Recent evidence indicates that there is a considerable inter-relationship between the immune and neuroendocrine systems.[1–7] A number of classical hormones, including steroids, peptides and proteins, are able to exert a wide variety of effects on immune responses, and receptors for these hormones are located on the lymphocytes. In turn, factors released from the immunocytes may influence endocrine systems.[5–7] Therefore, it may well be hypothesized that there may exist an immunoregulatory negative feedback circuit between immune and neuroendocrine systems. It has been shown that glucocorticoid inhibited the production of interleukin activity as measured by bioassay, in rats and mice. However, striking differences in susceptibility to glucocorticoids among species exist. Steroid-sensitive species including the mouse, rat, rabbit and hamster, experience dramatic lymphoid depletion after the treatment. In contrast, guinea-pig, monkey and human lymphocytes are resistant to the lytic effects of glucocorticoids.[8–10] Furthermore, the bioassay is known to be influenced by other lymphokines and monokines, and also the carry-over of glucocorticoids from the original cultures could influence the bioassay.

Therefore, we investigated the effect of glucocorticoid on IL-6 production in human peripheral blood mononuclear cells using a newly developed specific radioimmunoassay system for IL-6.

### Materials and Methods

*Subjects:* Normal subjects drawn from hospital volunteers (six male, four female: age range 23–56 years) were studied. No subjects were on medication or had any evident diseases.

*Materials:* Recombinant human IL-6 was purchased from Genzyme, Cambridge, MA, USA. Lipopolysaccharide (LPS s. typhosa 0901) and Concanavalin A (Con A) were obtained from Difco Laboratories,

© 1992 Rapid Communications of Oxford Ltd.

Detroit, MI, USA. Phytohemagglutinin (PHA) was purchased from GIBCO, New York, USA. Various hormones, peptides, lectins, cytokines and growth factors were obtained from the Protein Research Foundation, Osaka, Japan. $^{125}$I-recombinant IL-6 (specific activity: 5660 KBq/$\mu$g) was obtained from Amersham, England. Other substances were purchased from Sigma, USA.

*Immunization:* Anti-recombinant human IL-6 antibodies were generated in our laboratories as previously reported in a method for IL-2 antibody[11]. Two New Zealand white rabbits were immunized with 10 $\mu$g of IL-6 in 1.0 ml water emulsion in complete Freund's adjuvant using multiple subcutaneous injections at intervals of 4 weeks after each injection. The presence of anti-IL-6 was checked.

*Radioimmunoassay:* All assays were performed in duplicate in 12 × 75 mm polystyrene tubes. Duplicate 100 $\mu$l volumes of samples or standards were used and results were expressed as ng/ml of the original samples. For the assay, 0.01 M phosphate buffer (pH 7.4) with 0.15 M NaCl and 0.2% BSA was used. A double antibody radioimmunoassay was performed utilizing goat anti-rabbit gammaglobulin. A schematic diagram of the methodology was shown in Table 1.

*Culture of human peripheral blood mononuclear cells:* Blood from ten normal human subjects was separated on Ficoll–Hypaque and peripheral blood mononuclear cells (PBMC) were suspended in serum-free medium (Nihon Seiyaku Co., Japan) composed of various amino acids with insulin 2 mg/l, transferrin 2 mg/l, ethanolamine 0.122 mg/l, selenite 0.00914 mg/l being added 100 $\mu$g/ml streptomycin, 100 U/ml penicillin, 0.01 M HEPES buffer (GIBCO, USA) which had been passed through a ultramilipore filter. The cell concentration was 1 × $10^6$ cells/ml. Five hundred $\mu$l of the cell suspension was added to flat bottom microtiter wells (NUNC, Denmark) and 50 $\mu$l of either control medium or medium containing various substances (10 $\mu$g/ml of LPS and/or varying concentrations of hydrocortisone ranging from $10^{-10}$ M to $10^{-3}$ M) were added. After indicated hours of incubation at an atmosphere of 5% $CO_2$–95% air in a humidified tissue culture incubator at 37°C, at the end of 24, 48, 72 and 96 h, triplicate cultures were terminated by centrifugation and collection of supernatant fluids. Cell viability as assessed by trypan blue exclusion was always greater than 96%. And hydrocortisone itself did not affect the viability.

*Column chromatography:* Culture supernatant medium containing high IL-6 concentration was obtained by stimulating human peripheral blood cells and chromatographed on a Sephadex G-50 (1 × 60 cm)

**Table 1.** A schematic diagram of the assay procedure for IL-6

| | |
|---|---|
| standard or samples | 0.1 ml |
| antibody (1:800) | 0.1 ml |
| $^{125}$I-IL6 | 0.1 ml |
| buffer | 0.5 ml |

↓
incubated for 24 h at 4°C
↓
added second antibody solution 0.1 ml
↓
incubated for 24 h at 4°C
↓
cnetrifuged at 3000 rpm at 4°C
↓
decanted supernatants
↓
counted (precipitants)
↓
calculated bound/total count (B/T)

**Table 2.** Relative reactivity of cytokines, growth factors, hormones, peptides and lectins in IL-6 radioimmunoassay

| | relative reactivity* |
|---|---|
| human recombinant IL-6 | 100 |
| human recombinant IL-1α | <0.001 |
| human recombinant IL-1β | <0.001 |
| human recombinant IL-2 | <0.001 |
| human recombinant IL-3 | <0.001 |
| human recombinant IL-4 | <0.001 |
| human recombinant IL-5 | <0.001 |
| Interferon β | <0.001 |
| TNF | <0.001 |
| NGF | <0.001 |
| Insulin | <0.001 |
| TRH | <0.001 |
| LHRH | <0.001 |
| Somatostatin | <0.001 |
| GHRH | <0.001 |
| CRH | <0.001 |
| TSH | <0.001 |
| LH | <0.001 |
| FSH | <0.001 |
| ACTH | <0.001 |
| Prolactin | <0.001 |
| β-endorphin | <0.001 |
| β-casomorphin | <0.001 |
| Leucine-enkephalin | <0.001 |
| Methionine-enkephalin | <0.001 |
| Dynorphin 1–13 | <0.001 |
| Naloxone | <0.001 |
| VIP | <0.001 |
| Substance P | <0.001 |
| CCK-8 | <0.001 |
| Tuftsin | <0.001 |
| Triiodothyronine | <0.001 |
| Thyroxine | <0.001 |
| LPS | <0.001 |
| IgG sorb | <0.001 |
| PHA | <0.001 |
| Con A | <0.001 |
| PWM | <0.001 |
| *Staphylococcus aureus* Cowan 1 | <0.001 |
| Histamine | <0.001 |
| Serotonin | <0.001 |
| Dopamine | <0.001 |

* Arbitrary values of human recombinant IL-6 = 100

and eluted with 0.01 M phosphate buffer (pH 7.4) collecting 1.0 ml fractions. The column was calibrated with protein molecular weight markers. Human recombinant IL-6 dissolved in culture medium was similarly chromatographed. The recovery of IL-6 from the column was evaluated by addition of $^{125}$I-IL-6 and was found to be approximately 60%.

*Recovery study:* The known amounts of IL-6 were added to the culture medium. The samples were incubated at room temperature for 1 h before being assayed in the IL-6 radioimmunoassay.

*Statistics:* Mean and standard deviation of the mean were calculated. Student's *t*-test was used to evaluate the differences and it was considered significant when $p < 0.05$.

### Results

*Generation of antibodies to IL-6:* Both rabbits responded to the immunization developing antibodies to IL-6 at a final titer of 1:2 000 or higher. An antiserum used in this study was obtained 1 week after the fourth injection. This serum showed a specific binding at a final dilution of 1:16 000.

*Specificity of antiserum:* The specificity of anti-IL-6 is shown in Table 2. No crossreactivity was observed with cytokines, growth factors, various hormones, peptides and lectins (Table 2).

*Binding of $^{125}$I-IL-6 to anti-IL-6:* A comparison of the binding inhibition curves obtained from a series of three experiments when the known concentrations of IL-6 are diluted in either assay buffer or culture medium is shown in Fig. 1. The binding inhibition curves were virtually superimposable when $^{125}$I-recombinant IL-6 (Amersham, England) was used. The dilution curve drawn by culture supernatant medium containing high IL-6 level produced by PBMC was parallel with the standard curve (Fig. 1). 50 pg/ml of IL-6 was consistently detected in this assay system. Intra-assay and inter-assay variations were 6.9% and 7.2%, respectively, when assayed by eight identical samples.

*Column chromatography:* As depicted in Fig. 2, culture supernatant medium with a high IL-6 concentration produced by PBMC on Sephadex G-50 showed one

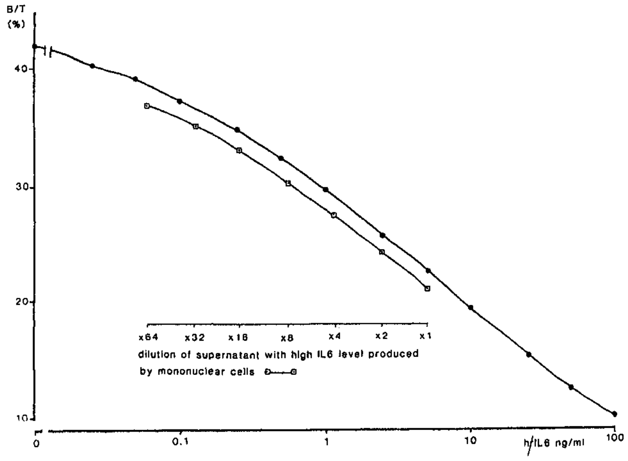

FIG. 1. Standard curves for the IL-6 radioimmunoassay with IL-6 standards diluted in buffer or culture medium (●—●) incubated with $^{125}$I-recombinant IL-6 and dilution curve by supernatant medium with high IL-6 concentration produced by stimulating human peripheral blood mononuclear cells (▣—▣).

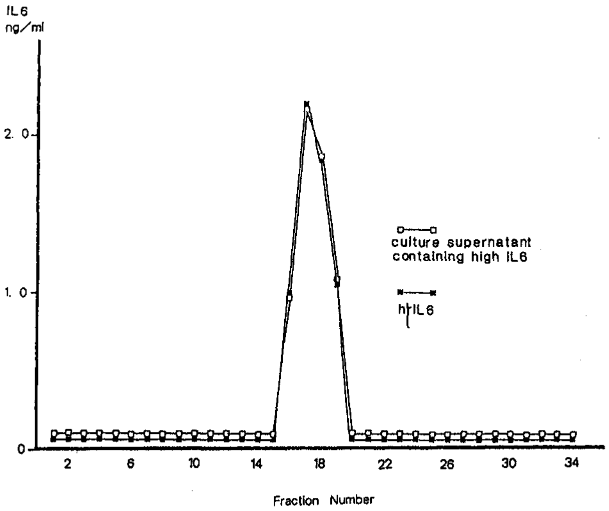

FIG. 2. Elution profile of authentic human recombinant IL-6 (■) and immunoreactive IL-6 produced by human peripheral blood mononuclear cells (□) on Sephadex G-50.

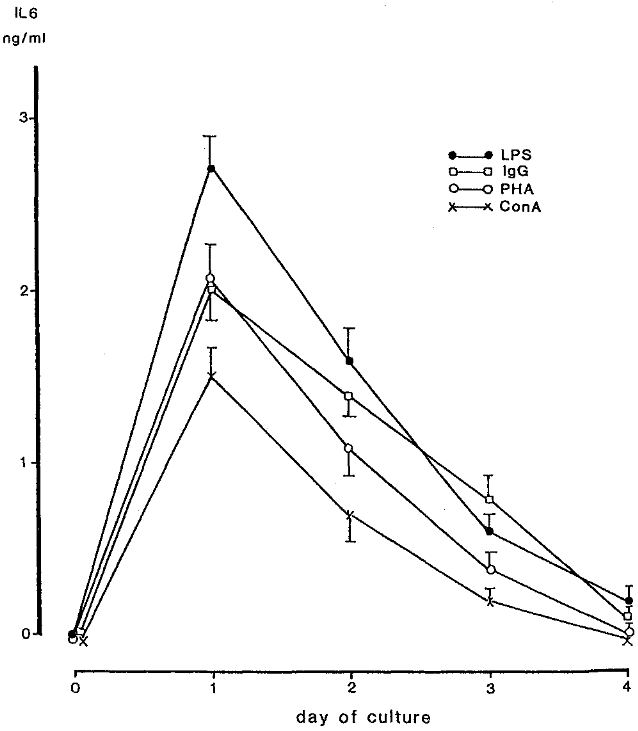

FIG. 3. Kinetics of IL-6 production. The Fig. shows IL-6 concentration measurable in supernatants of cultured human peripheral blood mononuclear cells. Each point represents mean ± SD of IL-6 level of supernatants in ng/ml, plotted on vertical axis ($n = 10$). Closed circles, squares, open circles and crosses represent LPS-, IgG-, PHA- and Con A-stimulated PBMC, respectively.

peak of immunoreactive material which corresponded to IL-6 (Fig. 2).

*Recovery of IL-6 added to medium:* There is a nearly complete recovery of IL-6 when added to medium (data not shown).

*Kinetics of IL-6 production:* Human PBMC from ten healthy normal subjects were set up in culture in microtitre plate. Cultures were initiated with or without LPS (10 μg/ml). IgG SORB(IgG, 0.1%), PHA (30 μg/ml) and Con A(100 μg/ml). At indicated times after the initiation (day 1, 2, 3 and 4) control and LPS-, IgG-, PHA- or Con A-stimulated cultures were terminated, the supernatants separated by centrifugation and assayed for IL-6, as shown in Fig. 3. The experiment was done three times and the results obtained were almost the same. Therefore the typical result was shown in Fig. 3. It was observed that detectable and peak levels of IL-6 were present in the LPS-, IgG-, PHA- and Con A-stimulated cultures after 24 h (mean $\pm$ SD; 2.71 $\pm$ 0.85 ng/ml, 1.99 $\pm$ 0.87 ng/ml, 2.06 $\pm$ 1.00 ng/ml and 1.50 $\pm$ 0.88 ng/ml, respectively) and then declined. The responses shown in Fig. 3 were maximal for each of the reagents tested. IL-6 levels were not detectable at any time in the supernatants of the corresponding eight unstimulated control cultures.

*Effect of hydrocortisone on IL-6 production:* Hydrocortisone alone did not raise IL-6 production at any of the concentrations tested. PBMCs were stimulated with LPS and cultured in the presence of hydrocortisone ($10^{-10}$ M to $10^{-3}$ M). IL-6 production by LPS-stimulated PBMC was found to be dramatically sensitive to the presence of hydrocortisone. As shown in Fig. 4, a dose-dependent hydrocortisone-induced inhibition of IL-6 production was observed. However, there was a wide variation in the response to hydrocortisone, with three distinct populations being identified. Based on the concentration required to inhibit 50% of LPS-stimulated IL-6 production, three of ten subjects were at $10^{-6}$ M, three at $10^{-5}$ M and the remaining four at $10^{-4}$ M (Fig. 4). At the concentration of $10^{-3}$ M, there was almost complete

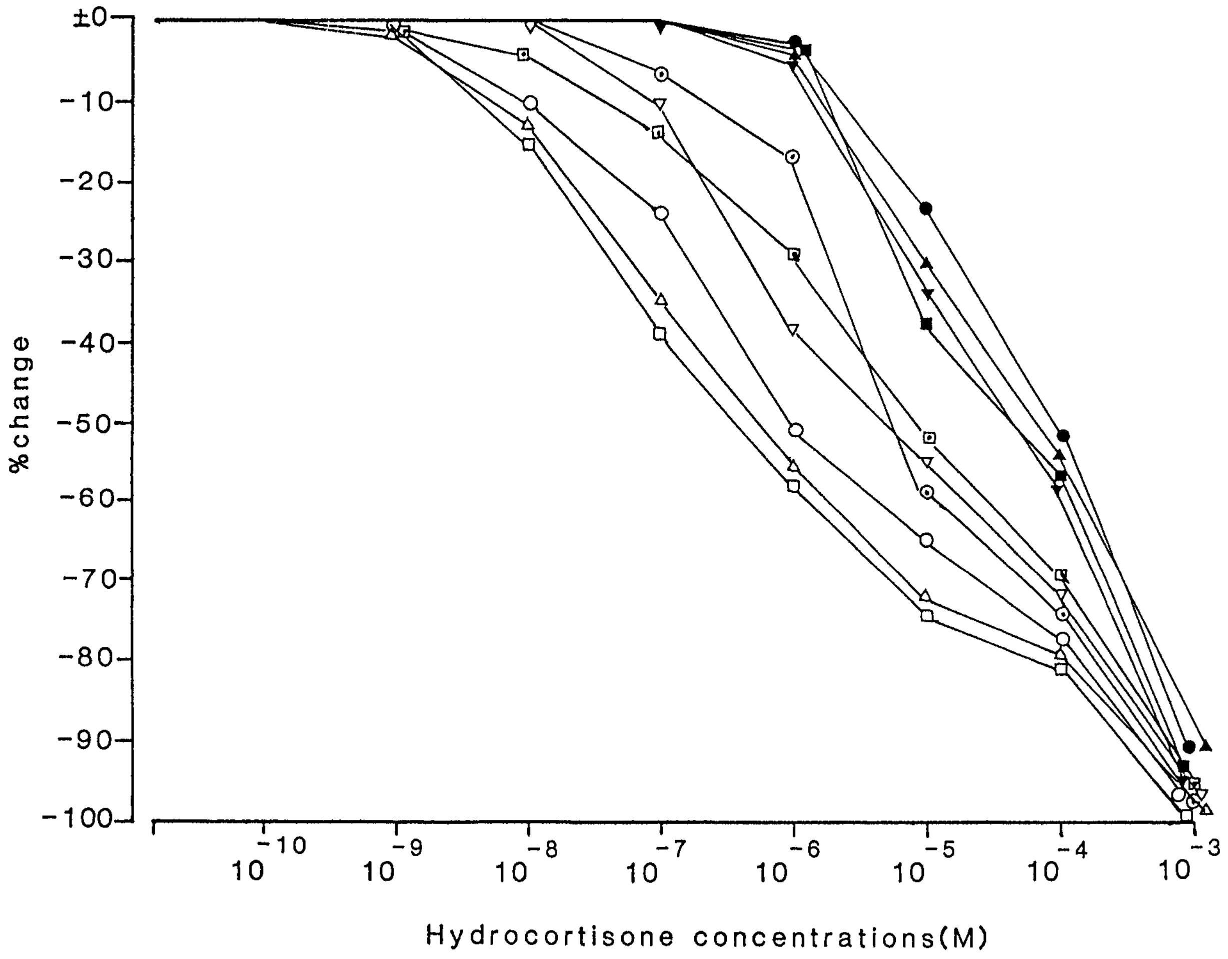

FIG. 4. Effects of varying concentrations of hydrocortisone ranging from $10^{-10}$ M to $10^{-3}$ M on IL-6 production stimulated by LPS (10 μg/ml) in ten normal human peripheral blood mononuclear cells. Each symbol represents an individual subject. SD of triplicate cultures was omitted to avoid further complication of the Fig. Without exception, the SD obtained at any cases was never greater than 8.7%. Results expressed as per cent decrease of the incubation without hydrocortisone.

inhibition of IL-6 production, whereas $10^{-10}$ M hydrocortisone produced IL-6 equal to that induced by LPS alone. The experiment was carried out on blood from the same individual three times and the results were almost the same.

### Discussion

We have developed a radioimmunoassay for human IL-6 with a sensitivity to 50 pg/ml. The assay utilized iodinated human recombinant IL-6 and rabbit antisera raised against IL-6. The assay detects IL-6 in the presence of mitogens or substances that might interfere with bioassay systems for IL-6. The specificity, reproducibility and usefulness have been demonstrated.

The immunosuppressive effect of glucocorticoid *in vivo* and *in vitro* is well documented and glucocorticoid is widely used in a variety of diseases for it. Today, increasing evidence suggest that there exists bidirectional communication between the immune and neuroendocrine systems. In the present study, we observed that hydrocortisone inhibited LPS-stimulated IL-6 production in a dose-dependent manner. Given the key role of IL-6 in enhancing lymphocyte function, hydrocortisone inhibition of IL-6 is likely to be an important immunosuppressive effect of this drug *in vivo*. It was also observed that there was a wide variation in the responsiveness to hydrocortisone in terms of the concentrations required to inhibit 50% of the LPS-stimulated IL-6 production. There was no discernable relationship between the inhibitory effect of hydrocortisone and age or sex in the present study. Some investigators suggested that activated lymphocytes were insensitive to glucocorticoid inhibition.[8,9] Our results are in disagreement with this, but, in agreement with the more recent report that indicated that activated lymphocytes contain increased numbers of glucocorticoid receptors and are sensitive to glucocorticoid-induced metabolic inhibition.[10] However, we cultured human peripheral blood mononuclear cells which contain not only lymphocytes but also monocytes in terms of the source of IL-6 production. Therefore, the experiments may not permit conclusions on the direct effects of glucocorticoids only on lymphocyte function. The varied inhibition of IL-6 production by glucocorticoid may partly explain the widely believed-notion that the human is a steroid resistant species,[8] although the physiological relevance of the effects is not clear because the effects were seen at pharmacological doses. The present findings suggested that a major mechanism of glucocorticoid-mediated immunosuppression may work at the level of the IL-6 production. And the data also suggested that to measure the glucocorticoid-sensitivity to IL-6 production *in vitro* might provide an indicator for the efficacy of the immunosuppressive therapy of steriods *in vivo*.

### References

1. Stein M, Chiavi RC, Camerino M. Influence of brain and behavior on the immune system. *Science* 1976; **191:** 435–440.
2. Riley V. Psychoneuroendocrine influences on immunocompetence and neoplasia. *Science* 1981; **212:** 1100–1109.
3. Keller SE, Weiss JM, Schliefer SJ, *et al.* Suppression of immunity by stress: effect of a graded series of stressors on lymphocyte stimulation in the rat. *Science* 1981; **213:** 1397–1399.
4. Blalock JE. Relationships between neuroendocrine hormones and lymphokines. In: E. Pick, Ed. *Lymphokines, vol. 9.* New York: Academic Press, 1984; 1–13.
5. Kelly KW. Cross-talk between the immune and endocrine-systems. *J Anim Sci* 1988; **66:** 2095–2108.
6. Dantzer R, Kelly KW. Stress and immunity: an integrated view of relationships between the brain and the immune system. *Life Sci* 1989; **44:** 1995–1008.
7. Blalock JE. A molecular basis for bidirectional communication between the immune and neuroendocrine systems. *Physiol Rev* 1989; **69:** 1–32.
8. Claman H. Corticosteroids and lymphoid cells. *N Engl J Med* 1972; **287:** 388–397.
9. Baxter JD, Harris AW. Mechanism of glucocorticoid action: general features, with respect to steroid-mediated immune suppression. *Trans Proc* 1975; **7:** 55–65.
10. Smith KA, Crabtree GR, Kennedy SJ, *et al.* Glucocorticoid receptors and sensitivity of mitogen stimulated and unstimulated lymphocytes. *Nature* 1977; **267:** 523–526.
11. Hirooka Y, Ohtake K, Nogimori T, *et al.* Impaired production of interleukin-2 (IL-2) in patients with Graves' Disease by newly developed Il-2 radioimmunoassay. *Exp Clin Endocrinol* 1990; **96:** 64–72.

**Received 30 September 1991;**
**accepted with revision 25 October 1991**
